# Virtual reality hypnosis prior to radiofrequency thermocoagulation for patients with chronic pain: an exploratory clinical trial

**DOI:** 10.3389/fpsyg.2024.1331826

**Published:** 2024-02-27

**Authors:** Othmane Safy, Floriane Rousseaux, Marie-Elisabeth Faymonville, Dominique Libbrecht, Robert Fontaine, Melissa Raaf, Cécile Staquet, Hadrien Tasset, Vincent Bonhomme, Audrey Vanhaudenhuyse, Aminata Bicego

**Affiliations:** ^1^Sensation and Perception Research Group, GIGA-Consciousness, University of Liège, Liège, Belgium; ^2^Medical Hypnosis Laboratory, MaisonNeuve-Rosemont Hospital Research Center, University of Montreal, Montreal, QC, Canada; ^3^Interdisciplinary Algology Center, University Hospital of Liège, Liège, Belgium; ^4^Oncology Integrated Arsen Bury Center, University Hospital of Liège, Liège, Belgium; ^5^Vivalia, Hospital of Libramont, Bastogne, Belgium; ^6^Department of Anesthesia and Algology, CHC Mont Legia, Liège, Belgium; ^7^Anesthesia and Perioperative Neuroscience Laboratory, GIGA-Consciousness, Liège University, Liège, Belgium; ^8^Department of Anesthesia and Intensive Care Medicine, Liège University Hospital, Liège, Belgium

**Keywords:** virtual reality, hypnosis, chronic pain, anxiety, radiofrequency thermocoagulation

## Abstract

**Background:**

The management of chronic pain may involve an array of tools, including radiofrequency thermocoagulation (Rf-Tc) of sensory nerve terminals. Like many other invasive procedures, Rf-Tc can generate anxiety in a lot of patients, either during the expectation of the procedure or in the course of it. Virtual reality hypnosis (VRH) is a promising tool for managing anxiety and pain in several situations, but its anxiolytic property has not been investigated in participants with chronic pain and going through a Rf-Tc procedure.

**Objectives:**

The goal of this study was to evaluate the effectiveness of VRH for reducing self-assessed anxiety in participants with chronic pain, when received in preparation for Rf-Tc.

**Materials and methods:**

This prospective, controlled trial was conducted in the Interdisciplinary Algology Centre of the University Hospital of Liège (Belgium). Participants were assigned to two groups: VRH or control (usual care). Assessment was carried-out at 4 time points: T0 (one week before Rf-Tc); T1 (pre-intervention, on the day of Rf-Tc); T2 (immediately after the VRH intervention outside of the Rf-Tc room); and T3 (right after Rf-Tc). Medical, sociodemographic data, anxiety trait and immersive tendencies were collected at T0. Anxiety state and pain intensity were assessed at each time points. Satisfaction was examined at T3.

**Results:**

Forty-two participants were quasi-randomly assigned to the VRH or control group. No statistically significant interaction group by time was observed regarding all measured variables, including primary endpoint. However, a significant effect of time was found for anxiety and pain when considering both groups together, toward a progressive reduction.

**Conclusion:**

In the context of our study, there appears to be no significant effect of VRH at reducing anxiety in participants with chronic pain undergoing Rf-Tc. Anxiety decreases along the procedure, while pain is attenuated by the local anesthetic infiltration of the Rf site. Our results suggest that the presence of a caregiver throughout the procedure might explain the progressive decrease in anxiety. Future randomized controlled trials are needed to precisely study the effectiveness of the VRH tool, and the possibility of using it as a complementary approach for anxiety during invasive procedures.

## 1 Introduction

Chronic pain (CP) is a complex phenomenon, characterized by persistent pain lasting at least 3 months (Turk et al., 2011), and involving biological, psychological, and socio-professional factors that impact patients’ global quality of life (Gatchel et al., 2007). Currently, negative affects (i.e., depression, anxiety, emotional distress, negative emotions) are the most assessed psychological parameters in CP, with evidence that it contributes significantly more than pain intensity to long-term outcomes of persistent pain such as physical and work disability, healthcare costs, mortality, and even suicide (Meints and Edwards, 2018). Even though several medications (e.g., non-steroidal anti-inflammatory drugs, opioids) are commonly used for the management of CP, these treatments often come with specific and well-documented negative side effects (Foster et al., 2018). They are usually recommended in conjunction with other approaches like physiotherapy, cryotherapy and psychotherapy, among others, falling within a biopsychosocial framework (Hylands-White et al., 2017).

Depending on the indication, invasive procedures can be proposed as first line treatment or when patients do not respond to conservative measures (Hylands-White et al., 2017). These procedures are known to trigger high levels of anxiety in concerned patients, both in relation to the anticipation of the event and during the procedure (Kindler et al., 2000). High levels of anxiety are known to impede quality of life, and to slow recovery down after an invasive procedure (Peters et al., 2007). In our study, we focused on radiofrequency thermocoagulation (Rf-Tc) of the sensitive innervation of the spine’s facet joints.

Rf-Tc is an invasive procedure that blocks the transmission of nociceptive information from peripheral receptors to the central nervous system by damaging nerve fibers in a targeted nervous structure using heat (Pevsner et al., 2003). In patients suffering from a facet syndrome at the cervical, thoracic, or lumbar level, that is, pain related to osteoarthritis of those joints, Rf-Tc is effective at reducing pain for periods of 4 to 6 months. In our population, and in addition to osteoarthritis, Rf-Tc was also proposed for relieving other types of pains such as chronic coccydynia or non-osteoarthritic causes of chronic low back pain (e.g., herniated disk, compression fracture).

Recently, there has been growing interest for a new complementary approach combining hypnosis and virtual reality (VR) in various clinical contexts (Rousseaux et al., 2020), a technique called virtual reality hypnosis (VRH). Hypnosis is an effective intervention to reduce pain perception, depression, and anxiety, while also improving quality of life in patients with CP (Bicego et al., 2021). Hypnosis is defined as “a state of consciousness involving focused attention and reduced peripheral awareness, characterized by an enhanced capacity for response to suggestion”(Elkins et al., 2015). VR is a technology that immerses individuals by providing them with a sense of presence in a three-dimensional (3D) computer-generated world or virtual environment, that can be explored interactively using variable peripheral computer devices (Aziz, 2018). VRH can be described as the delivery of hypnotic induction and suggestions by customized VR hardware/software (Patterson et al., 2004). The interest of combining hypnosis and VR, as compared to VR alone or to hypnosis delivered by an external care giver, is to use a virtual 3D environment to immerse patients, while they are guided by hypnotic suggestions at the same time (Rousseaux et al., 2020). The interest of VRH for the improvement of patients’ comfort has been evaluated in different medical contexts, such as trauma, pneumology or intensive care (Patterson et al., 2004; Lachkar et al., 2022; Rousseaux et al., 2022a). The present research focused on whether VRH can alleviate anxiety associated with an invasive procedure. While the participants in this study suffer from chronic pain, the aim is not to assess if VRH decreases chronic pain itself but rather to make a procedure designed for chronic pain relief more tolerable for patients (i.e., reduce anxiety). Thus, the primary goal of this study was to evaluate the effectiveness of VRH at reducing self-assessed anxiety in CP participants having to undergo Rf-Tc.

## 2 Methods

### 2.1 Population

From March 1st, 2021, to March 31st, 2022, participants with CP were recruited when they attended the Algology Interdisciplinary Center of the University Hospital of Liège (Belgium) to receive a Rf-Tc procedure. The inclusion criteria were: participants suffering from CP, being aged >18 years, being French speaking, having no claustrophobia, having no head or face wounds, having sufficient auditory and visual acuity for an effective use of the VRH technique. Participants were referred to the study if they had received an indication for Rf-Tc by an algologist, physical therapist, rheumatologist, or neurosurgeon. Thirty-eight participants were randomized into two groups: a control group who benefited from usual care (CTR; *n* = 15) and an experimental group who benefited from VRH (*n* = 18). In the CTR group, 6 participants were withdrawn from the study because of technical issues. We thus decided to add 4 additional participants to the CTR group to be faithful to the sample size calculation.

### 2.2 Ethical consideration

The study was approved by the Ethics Committee of the Faculty of Medicine of the University of Liège, Belgium (reference number: 2020–344), and was in accordance with the General Data Protection Regulation and with the 1964 Helsinki declaration and its later amendments. The study was retrospectively registered on ClinicalTrials.gov (number: NCT06082427). All participants gave written informed consent to participate in the study.

### 2.3 Procedure

The study was a prospective, quasi-randomized controlled trial which subsequently underwent a design modification as some participants (*n* = 4) were added without being randomized (see section 2.1 for more details). Except for these 4 participants, all other volunteers were randomized into two groups (with the randomization function of Microsoft Excel): a CTR group and a VRH group. The procedure included four phases. Every participant scheduled for Rf-Tc was contacted by telephone to propose them to participate in the study. This first step occurred one week before Rf-Tc and consisted in a screening phase, in which the study protocol was explained, and verbal consent was asked to participants, prior to written consent. At that time, socio-demographic data were recorded, and anxiety trait (Spielberger et al., 1983), propensity to immersion (Witmer and Singer, 1998), anxiety intensity (Benotsch et al., 2000) and pain intensity (Bijur et al., 2001) were evaluated (T0). The second step occurred on the day of the Rf-Tc procedure. On that day, participants were first invited to lay comfortably on a hospital bed, and anxiety (Benotsch et al., 2000) and pain (Bijur et al., 2001) intensity were assessed (T1). Then, participants assigned to the VRH group benefited from 17 min of VRH, while patients from the CTR group were asked to relax and wait with no distractions during 17 min. Immediately after this 17-min period, anxiety intensity (Benotsch et al., 2000) and pain intensity (Bijur et al., 2001) were again assessed (T2). The third step was the Rf-Tc procedure which was applied on the spinal facet joints of either the cervical, thoracic, lumbar, or sacrococcygeal region and ganglion impar. Right after the procedure, anxiety intensity, pain intensity and satisfaction were assessed (T3).

### 2.4 Material

#### 2.4.1 Intervention

**Figure 1:**
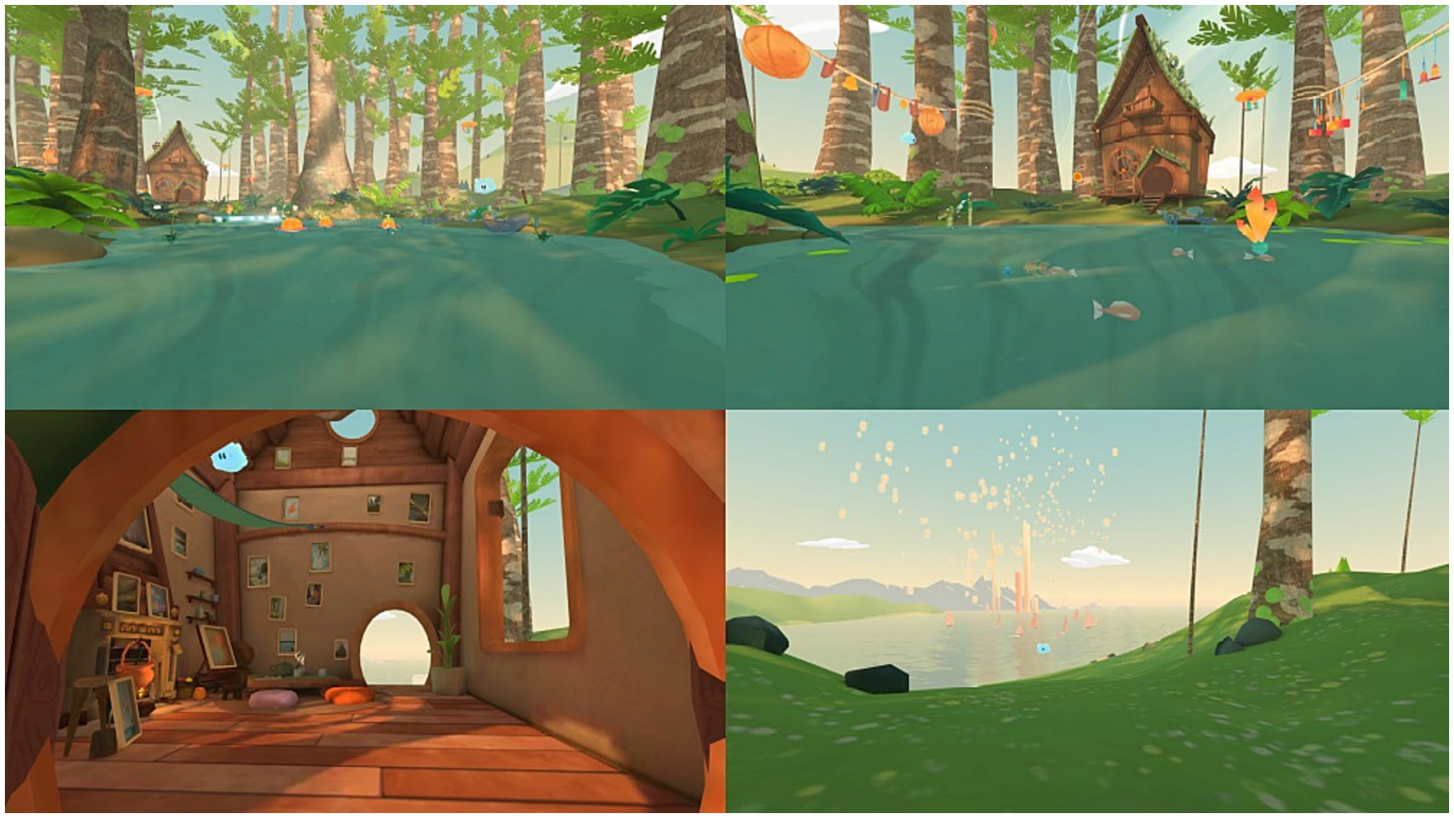
Illustration of four scenes that participants can see during the virtual reality hypnosis experience. ^©^The Lanterns Wood – IPNEO, designed by the society Cayceo (Montpellier, France, https://cayceo.fr/).

VRH was delivered through a Pico G2 4K virtual reality headset equipped with a head-tracking system and the « IPNEO » software designed by Cayceo (Montpellier, France).^1^ IPNEO is a certified software medical device displaying an enchanted 3D animated environment called « The Lanterns Wood ». The script of the software allows hypnosis induction and suggestions (relaxation, comfort, and safety). When the immersive experience begins, the participants find themselves on a platform placed on a river, and slowly move toward a wooden hut. The environment consists of trees, fireflies, luminous red ball, a river, as well as various silhouettes of animals (Figure 1). During the session, a male voice invites the participants to relax, enjoy the moment and focus on the present moment by suggesting pleasant sensations. The trip continues until the participants enter a hut, where they discover a unique decor (frames, windows, fireplace, etc.). The participants stay inside for 1 min and 40 s, while the narrator continues to deliver positive suggestions. The intervention ends when the participants leave the hut and find themselves surrounded by trees. The participants are then brought back to the “here and now” and are given post-hypnotic suggestions to maintain the calm and relaxation they have experienced during the VRH. The complete intervention lasts 17 min.

#### 2.4.2 Self-reported measures

*The recorded medical and socio-demographic data* were age, sex, nationality, level of education, socio-professional, marital status, type and location of Rf-Tc, previous Rf-Tc, previous experience in VR and/or hypnosis, diagnosis, pain duration, and current medical treatment.

*The State–Trait Anxiety Inventory (STAI - Y)* (Spielberger et al., 1983) was used to assess trait anxiety only. Originally this questionnaire has two parts one dedicated to assess state anxiety and another to assess the trait anxiety. Only the latter was used in this study. STAI – Y Trait contains 20 items with 4 response options (1 = almost never, 2 = sometimes, 3 = often, 4 = almost always). Total scores can range from 20 to 80, with higher scores indicating a higher level of anxiety. STAI – Y Trait was administered at T0 and the validated French version was used (Gauthier and Bouchard, 1993).

*The Immersive Tendencies Questionnaire (ITQ)* (Witmer and Singer, 1998) contains 18 items rated on a scale of 1 (never) to 7 (often). This questionnaire measures participant’s tendencies to immerse themselves or get involved in a virtual experience. It contains 4 sub-scales: “Focus” is the tendency to stay focused on ongoing activities (total score between 5 and 35); “Involvement” is the tendency to become involved in activities (total score between 5 and 35); “Emotion” is the tendency to be emotionally involved by the environment (total score between 4 and 28); “Game” is the tendency to play video games (total score between 3 and 21). A total score is also available, and can vary from 18 to 126. The higher the score, the higher the tendency for immersion in the virtual environment. The French version of the University of Quebec Outaouais Cyberpsychology Lab was used (Robillard et al., 2002).

*The Numerical Rating Scale (NRS)* (Benotsch et al., 2000; Bijur et al., 2001) is a self-assessed scale that ranges from 0 to 10. Three French versions were used to assess anxiety intensity (0 = no anxiety, 10 = the most intense anxiety), pain intensity (0 = no pain, 10 = the most intense pain imaginable) and satisfaction about the procedure (0 = total dissatisfaction, 10 = total satisfaction). Participants had to answer according to the present moment.

### 2.5 Sample size

The sample size calculation was based on a repeated measure analyses of variance (ANOVA) within – between interaction. Alpha was set at 0.05, power at 95% and the effect size at 0.5. According to this analysis, 19 participants were required in each group for a total of 38 participants.

### 2.6 Statistical analysis

First, descriptive statistics were conducted. Qualitative variables were expressed with count and percentage. If normality was assumed for the distribution of the quantitative variable, means and standard deviations were reported. Reversely, medians and interquartile ranges were presented. Normality of the data was evaluated by comparing mean and median, graphically using a histogram and a quantile-quantile plot, and by carrying out a Shapiro–Wilk test. To detect potential confounding factors, baseline characteristics were compared between the 2 groups using χ2 test for qualitative variables and Student *t*-test or its equivalent non-parametric test, namely the Mann–Whitney U test, for quantitative variables. Repeated measures analyses of variance (ANOVA) time x group were applied to examine the evolution of anxiety (NRS), and pain intensity (NRS) across the 4 time points of interest (T0, T1, T2, T3) and between the 2 groups (CTR and VRH). Effect sizes were also calculated for both anxiety and pain (NRS). Results were considered significant at the 5% critical level (two-tailed *p* < 0.05). Multiple comparisons using Bonferroni as correction method were conducted to assess the evolution of both anxiety and pain over time with adjusted *p* values. The analyses were conducted with the software Jamovi version 2.3.21 (Project, J, 2022).

## 3 Results

### 3.1 Study population

Out of the 53 approached participants, ten refused to participate in the study due to lack of motivation (i.e., the patients said to be not motivated in participating in the study). One participant canceled the Rf-Tc appointment, so dropped-out from the start, and 4 participants did not meet the inclusion criteria (impaired audition *n* = 2, not fluent in French *n* = 2). The remaining 38 participants were randomized into two groups: a CTR group (*n* = 17) and a VRH group (*n* = 21). In the CTR group, 6 participants were withdrawn from the study because of technical issues. We thus decided to add 4 additional participants in the CTR group to be faithful to the sample size calculation. Thus, these participants were not randomized, and that explains the quasi-randomization in this study. Out of the 21 participants in the VRH group, 3 dropped-out for different reasons (2 participants canceled their Rf-Tc appointment, and one dropped-out because of cybersickness following the VRH intervention). In total, the CTR group was composed of 15 participants and the VRH group of 18 participants.

### 3.2 Descriptive analysis

The participants included in the analysis consisted in 18 women and 15 men. Their age was 58.4 (14.8) years [mean (SD)]. No statistical differences were observed between groups for age, sex, nationality, level of education, socio-professional situation, family situation, type of Rf-Tc, previous Rf-Tc, previous experience with VR and/or hypnosis, diagnosis, and pain duration. No statistical differences were observed for the total scores of the STAI-Trait (Gauthier and Bouchard, 1993) and the ITQ (Robillard et al., 2002) (see Table 1).

**Table 1:** Participants’ medical and socio-demographic characteristics for the global sample and within each group (CTR and VRH).

	Total sample (*N* = 33)	CTR group (*N* = 15)	VRH group (*N* = 18)	*p*-value
*Age (years), mean ± SD*	58.4 ± 14.8	58.9 ± 16.8	58 ± 13.4	0.86
*Sex, n (%)*				
Female	18 (54)	10 (30)	8 (24)	0.2
Male	15 (45)	5 (15)	10 (30)	
*Nationality, n (%)*				
Belgian	32 (97)	15 (46)	17 (51)	0.35
Italian	1 (3)	0	1 (3)	
*Educational level, n (%)*				
Secondary	25 (76)	12 (36)	13 (40)	0.6
Higher education	8 (24)	3 (9)	5 (15)	
*Marital status, n (%)*				
Single	7 (21)	4 (12)	3 (9)	0.5
Married	16 (48)	5 (15)	11 (33)	
Cohabiting	4 (12)	2 (6)	2 (6)	
Widow	1 (3)	1 (3)	0	
Divorced	5 (15)	3 (9)	2 (6)	
*Occupational status, n (%)*				
Employed	8 (24)	2 (6)	6 (18)	0.16
Unemployed/Disabled	11 (33)	4 (12)	7 (21)	
Retired	14 (42)	9 (27)	5 (15)	
*Types of Rf-Tc, n (%)*				
Lumbar	26 (79)	12 (37)	14 (42)	0.24
Cervical	3 (9)	0	3 (9)	
Impar ganglion	3 (9)	2 (6)	1 (3)	
Sacro-iliac	1 (3)	1 (3)	0	
*Diagnosis, n (%)*				
Back pain	28 (85)	13 (40)	15 (45)	0.3
Cervical pain	2 (6)	0	2 (6)	
Coccydynia	1 (3)	1 (3)	0	
Perineal pain	1 (3)	1 (3)	0	
Joint pain	1 (3)	0	1 (3)	
*Pain duration (years), mean ± SD*	14.5 ± 13.9	16.7 ± 17	12.8 ± 11.1	0.44
*VR previous experience, n (%)*				
Yes	9 (27)	5 (15)	4 (12)	0.48
No	24 (72)	10 (30)	14 (42)	
*Hypnosis previous experience, n (%)*				
Yes	10 (30)	5 (15)	5 (15)	0.73
No	23 (70)	10 (30)	13 (40)	
*Rf-Tc previous intervention, n (%)*				
Yes	16 (48)	9 (27)	7 (21)	0.23
No	17 (51)	6 (18)	11 (33)	
*Anxiety trait (20–80), mean ± SD*	39.6 ± 10.1	41.2 ± 9.3	38.3 ± 10.8	0.43
*Immersion tendencies (1–7), mean ± SD*				
Focus	25.2 ± 4.1	25.3 ± 4.3	25.1 ± 4.04	0.88
Involvement	16 ± 6.3	14.9 ± 6.5	16.9 ± 6.2	0.38
Emotion	11.1 ± 5.4	10.5 ± 4.3	11.6 ± 6.2	0.55
Game	7.6 ± 5.3	8.1 ± 6	7.2 ± 4.7	0.61
Total score	59.8 ± 14.4	58.8 ± 15.8	60 ± 13.5	0.71

CTR, control group; VRH, Virtual reality hypnosis group; SD, Standard deviation; Rf-Tc, Radiofrequency Thermocoagulation; VR, Virtual Reality.

### 3.3 Effect of the interaction group by time, time, and group

**Figure 2:**
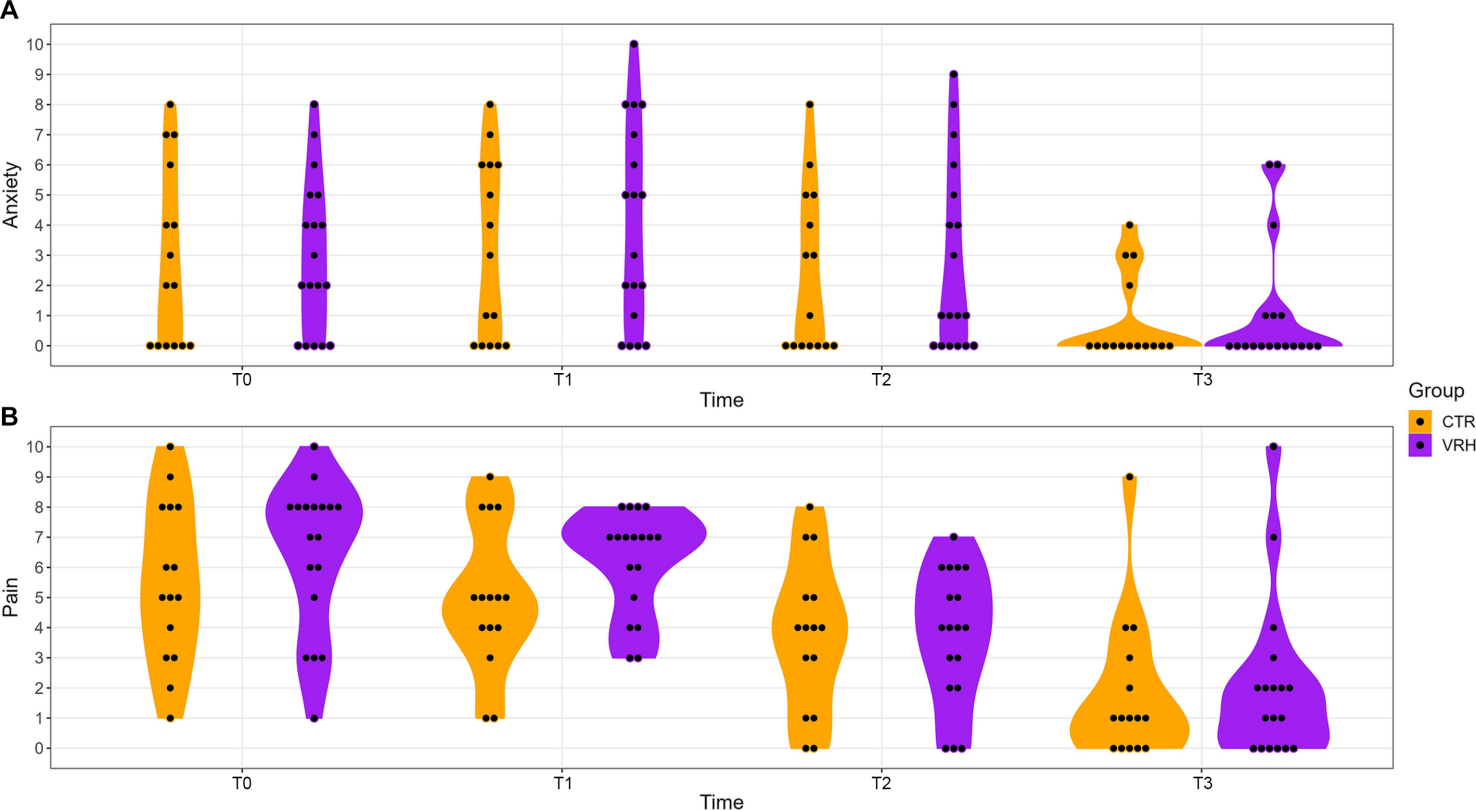
Evolution of anxiety and pain in both groups over time. Pannel **(A)** displays anxiety scores assessed via a numerical rating scale (0–10) at each time points. Pannel **(B)** shows pain intensity scores assessed via a numerical rating scale (0–10) at each time points. Purple color represents the VRH group while the orange represents the CTR group. VRH, Virtual reality hypnosis; CTR, control.

Concerning anxiety, no significant interaction group by time (*F* = 0.249, *p* = 0.86) and no group effect (*F* = 0.308, *p* = 0.58) were observed. Nevertheless, a significant main effect of time (*F* = 12.252, *p* < 0.001) was found. The same pattern of result was observed regarding pain intensity. Indeed, no significant interaction group by time (*F* = 0.749, *p* = 0.52) and no group effect (*F* = 0.946, *p* = 0.34) while a significant main effect of time (*F* = 32.327, *p* < 0.001) was found. Results show a decrease in anxiety and pain intensity over time. Effect size were small both for anxiety (η^2^ = 0.3) and pain intensity (η^2^ = 0.5) (see Table 2 and Figure 2).

**Table 2:** Evolution over time of mean and standard deviation (SD) of the primary and secondary outcomes in the CTR group and in the VRH group.

	CTR (*n* = 15)				VRH (*n* = 18)				Time	Effect size
Questionnaires (Mean ± SD)	T0	T1	T2	T3	T0	T1	T2	T3	*p*-value	η^2^
Anxiety (0–10, NRS)	2.9 ± 3	3.13 ± 3	2.3 ± 2.7	0.8 ± 1.4	3 ± 2.5	4 ± 3.3	2.8 ± 3.1	1.1 ± 2	<0.001	0.3
Pain (0–10, NRS)	5.5 ± 2.7	5 ± 2.4	3.7 ± 2.5	1.8 ± 2.4	6.4 ± 2.5	6.2 ± 1.7	3.7 ± 2.2	2.1 ± 2.7	<0.001	0.5
Satisfaction (0–10, NRS)	NA	NA	NA	8.9 + 1.3	NA	NA	NA	9.1 ± 1.3	0.66*	NA

The *p*-values and effect size concern the time effect only. T0 = screening by phone call; T1, pre-CTR/VRH; T2, post-CTR/VRH; T3, post-Tf-Rc; CTR, control; VRH, Virtual reality hypnosis; NRS, Numerical Rating Scale; NA, non-applicable; * the statistical comparison has been tested with a Mann–Whitney *U* test.

Post-hoc comparisons revealed that, over time, there was a significant decrease in anxiety between T0 and T3 (*p*^adj^ = 0.002), T1 and T2 (*p*^adj^ = 0.005), T1 and T3 (*p*^adj^ < 0.001), and T2 and T3 (*p*^adj^ = 0.004). Over time, a significant decrease in pain intensity was observed between T0 and T2 (*p*^adj^ < 0.001), T0 and T3 (*p*^adj^ < 0.001), T1 and T2 (*p*^adj^ < 0.001), T1 and T3 (*p*^adj^ < 0.001), and T2 and T3 (*p*^adj^ = 0.004). No significant difference was observed for anxiety (*t* = −0.14, *p* = 0.89) and pain (*t* = −1.02, *p* = 0.32) between the two groups at T0.

## 4 Discussion

The aim of this study was to evaluate the effectiveness of VRH on self-assessed anxiety as a primary outcome and pain intensity as a secondary outcome for participants suffering from CP and having to undergo a Rf-Tc. Globally, the participation rate in this study was good, with only 10 refusals out of 53 patients initially contacted and the mean satisfaction level was close to 9 out of 10 for both CTR and VRH groups. While a main effect of time for anxiety and pain intensity was observed, no significant interaction between time and group was found. Indeed, there was a significant decrease of both anxiety and pain intensity over time when considering all patients together.

Results coming from other studies have shown that VRH decreases anxiety and pain in the context of a medical procedure or a surgery which diverge from the present results. Lachkar et al. (2022) proposed VRH to 20 participants having to undergo a bronchoscopy (with local anesthesia). The VRH device displayed slow motion movies from various natural landscapes alongside headphones transmitting a narrative of hypnosis with sequences of controlled breathing, cardiac coherence and hypnotic suggestions. Results indicated a reduction of anxiety in all participants. In a prospective study on a group of 48 participants undergoing hand surgery (Touil et al., 2021), a 15-min VRH session was proposed after administering an axillary plexus block, while preparing the participants for surgery. The VRH device combined imagery, sounds and a narrative clinical hypnosis script with progressive muscle relaxation and deep breathing suggestions with a soothing music background. The results showed a significant decrease in anxiety scores following the VRH when compared to the anxiety scores prior to the VRH. Nevertheless, no control groups were included in these studies preventing to draw any firm conclusions. However, a randomized controlled trial, including 100 participants scheduled for peripheral endovascular interventions (under local anesthesia), found similar results as Touil et al. (2021), Lachkar et al. (2022), and Gullo et al. (2023). Finally, the only study assessing anxiety reduction in participants with CP having to undergo fluoroscopy-guided lumbar sympathetic ganglion block also showed that the VRH group had greater anxiety decrease than the control group (Joo et al., 2021). Noteworthy, in all of the above-mentioned studies, the VRH was proposed during the interventions. This might explain the discrepancy observed between the results of this study and the other ones. Indeed, while we acknowledge that the waiting prior for the intervention might be anxiogenic maybe proposing the device during the procedure might be more effective in this context of care.

In this study, baseline anxiety assessed at T0 and T1 was low in both groups depicting a low-anxiety sample whose reduction would probably not have contributed to a statistical interaction. Moreover, according to the STAI-Y, trait anxiety is considered as low when the total score is <52 for women and < 51 for men. This was the case in the participants included in this study. Perhaps offering VRH to participants with high trait or state anxiety would benefit them more compared to those with low levels of anxiety. Another hypothesis could be that participants in our study were simply relieved that the procedure was over, which would explain the absence of an interaction effect. Thus, screening participants based on their anxiety trait and/or state before a Rf-Tc could be a way to go for future studies.

Furthermore, the absence of a significant interaction effect could be that the participants received a different support than usually. The investigator phoned them before the intervention, welcomed them before the procedure and followed them through their stay probably representing a reassuring figure. This might have positively impacted their anxiety and the way they answered questionnaires whether they experienced VRH or not. This can be parallel with a previous study using VRH among 100 randomized cardiac surgery participants (Rousseaux et al., 2022a,b). Results showed no significant differences in anxiety from a presurgical phase to a postsurgical phase in the VRH group as compare to hypnosis alone, VR alone and a control group (Rousseaux et al., 2022a). Additionally, a study conducted with participants suffering from irritable bowel syndrome randomized into 3 groups (N = 262): placebo acupuncture alone, placebo acupuncture with a well-established patient-practitioner relationship and waiting list control group showed that an enhanced relationship with a practitioner, together with the placebo treatment, provides the most robust effect in terms of the four measures used in the study (i.e., global improvement, adequate relief, symptom severity, and quality of life) (Kaptchuk et al., 2008). In fact, empathy is a key feature to create insight into participants’ experience as if they were experiencing it themselves. Indeed, empathetic clinicians are able to communicate their understanding of the patient, both verbally and non-verbally, which can be therapeutic in itself (Rakel et al., 2011). Consequently, the presence of an investigator at every step of the procedure in our study may have contributed to reduce anxiety, as participants may have viewed that investigator as a reassuring empathetic figure or provider. Moreover, participants mentioned in several studies that the absence of companions or relatives, undergoing a procedure for the first time, lack of information, and waiting time before the procedure are all determinants of anxiety (Kaptchuk, 2002; Uzun et al., 2008; Vural et al., 2009). Thus the connection between participants and practitioners can impact the health status of participants by acting as a fundamental connection and providing social support (Kaplan et al., 1989; Roter and Hall, 1995). Another hypothesis could be purely statistical, indeed, it could be possible to have done a type 2 error wrongfully accepting the null hypothesis (equality between means). Nevertheless, when looking at the means’ evolution through the procedure, we can observe a similar decrease in both groups. Regarding pain and knowing that the site of Rf-Tc was anesthetized by infiltration with a local anesthetic agent mixture, a global decrease in pain intensity was expected. This supports the present findings.

The majority of the research on VR(H) aim at assessing their effectiveness in various clinical conditions while their processes remain understudied (Ioannou et al., 2020; Rousseaux et al., 2020). Regarding VR alone, it is hypothesized that distraction is the central mechanism behind it analgesic and anxiolytic effects it provides (Mahrer and Gold, 2009). Pain and anxiety capture attention so that the focus is on both of them. Through immersion, VR distracts attention from pain and/or anxiety leading to a reduction of both (Mahrer and Gold, 2009; Gupta et al., 2018). Potential mechanisms of action concerning VRH remain an open question. From its very beginnings, hypnosis has always been closely linked to dissociation. Dissociation can be defined as the “split off” of mental processes and bodily awareness and perceptions. Recently, a study using VRH highlighted that decreases in pain perception were negatively correlated to dissociation (Rousseaux et al., 2022b). Thus, dissociation might account for the analgesic and anxiolytic effect of VRH. Future studies should address processes at play in VR(H).

This study has some limitations. First, the design underwent some modifications due to technical issues altering the randomization. This could have influenced the overall results. Second, neither the participants nor the medical staff and the investigator were blind concerning the given intervention, because the motivation of the medical team and patients to use the tool is essential. Third, due to the 3 participants who dropped-out out from the VRH group and the 6 participants in the CTR group that were withdrawn from our analysis, it is possible that our results are due to underpowered statistics. Forth, some participants relied on the investigator to read and answer the questions, which could cause social desirability bias (Lemaine, 1965). To limit this issue, the investigator stayed as neutral as possible. Future studies should consider the therapeutic relationship and include the investigator as a variable, which could be assessed using therapeutic alliance scales like for example: Working Alliance/Theory of Change Inventory (WATOCI) (Hall et al., 2012) or Kim alliance scale (Kim et al., 2001).

## 5 Conclusion

Patients with chronic pain undergoing an invasive procedure like Rf-Tc can experience anxiety before, during and after the medical procedure. Despite a medical effort in finding adequate solutions, pharmacological agents can present some risk for some patients necessitating a personalized care. Complementary approaches such as VRH seem to provide anxiolytic effects when proposed in experimental and clinical settings. Unfortunately, the present findings could not demonstrate the latter assumption. Our results suggest that the presence of a caregiver throughout the procedure might explain the decrease in anxiety. Future randomized controlled trials are needed to precisely study the efficiency of the VRH tool, and the possibility of using it as a complementary treatment for anxiety during invasive procedures. While the use of VRH appears promising with regard to other studies, it is essential to consider the patient, the context and the timing in which it is applied and also consider the therapeutic relation as the basement of these interventions.

## Data availability statement

The raw data supporting the conclusions of this article will be made available by the authors, without undue reservation.

## Ethics statement

The studies involving humans were approved by Ethics Committee of the Faculty of Medicine of the University of Liège, Belgium. The studies were conducted in accordance with the local legislation and institutional requirements. The participants provided their written informed consent to participate in this study.

## Author contributions

OS: Formal analysis, Writing – original draft. FR: Conceptualization, Methodology, Writing – review & editing. M-EF: Conceptualization, Methodology, Resources, Writing – review & editing. DL: Resources, Writing – review & editing. RF: Resources, Writing – review & editing. CS: Resources, Writing – review & editing. HT: Writing – review & editing, Resources. VB: Writing – review & editing. AV: Supervision, Writing – original draft, Writing – review & editing, Conceptualization, Data curation, Funding acquisition, Investigation, Methodology, Project administration, Validation. AB: Supervision, Writing – original draft, Writing – review & editing, Formal analysis, Data curation, Investigation.

## Footnotes

^1^ https://cayceo.fr/
